# Membranes for Cation Transport Based on Dendronized Poly(epichlorohydrin-co-ethylene oxide). Part 1: The Effect of Dendron Amount and Column Orientation on Copolymer Mobility

**DOI:** 10.3390/polym13203532

**Published:** 2021-10-14

**Authors:** Alireza Zare, Borja Pascual-Jose, Silvia De la Flor, Amparo Ribes-Greus, Xavier Montané, José Antonio Reina, Marta Giamberini

**Affiliations:** 1 Department of Chemical Engineering, Universitat Rovira i Virgili (URV), Av. Països Catalans, 26, 43007 Tarragona, Spain; alireza.zare@urv.cat; 2 Institute of Technology of Materials (ITM), Universitat Politècnica de València (UPV), Camí de Vera, s/n, 46022 València, Spain; borpasjo@outlook.com (B.P.-J.); aribes@ter.upv.es (A.R.-G.); 3 Department of Mechanical Engineering, Universitat Rovira i Virgili (URV), Av. Països Catalans, 26, 43007 Tarragona, Spain; silvia.delaflor@urv.cat; 4 Department of Analytical Chemistry and Organic Chemistry, Universitat Rovira i Virgili (URV), C/Marcel.lí Domingo s/n, 43007 Tarragona, Spain; joseantonio.reina@urv.cat

**Keywords:** Poly(epichlorohydrin-co-ethylene oxide), dendrons, cation-transport membranes, self-assembly, membrane orientation, dynamic mechanical thermal analysis, dielectric thermal analysis

## Abstract

Dendronized polyethers give rise to columnar LC structures which can successfully act as cation transport materials. Therefore, we prepared two different materials, based on Poly(epichlorohydrin-co-ethylene oxide) (PECH-co-EO) grafted with methyl 3,4,5-tris[4-(n-dodecan-1-yloxy)benzyloxy] benzoate, containing 20% or 40% modified units, respectively. The obtained polymers were characterized by differential scanning calorimetry (DSC), X-ray diffraction and optical microscopy between crossed polars (POM) and compared to the unmodified PECH-co-EO. In order to reach efficient transport properties, homeotropically oriented membranes were prepared by a fine-tuned thermal annealing treatment and were subsequently investigated by dynamic mechanical thermal analysis (DMTA) and dielectric thermal analysis (DETA). We found that the presence of the dendrons induces a main chain partial crystallization of the polyether chain and coherently increases the polymer Tg. This effect is more evident in the oriented membranes. As for copolymer orientation upon annealing, the cooling rate and the annealing temperature were the most crucial factors. DMTA and DETA confirmed that grafting with the dendron strongly hinders copolymer motions, but did not show great differences between unoriented and oriented membranes, regardless of the amount of dendrons.

## 1. Introduction

Since Daniel Vorländer described in 1923 the synthesis of the first polymers that contains benzenes connected by ester bonds in the main chain [1], LCP (liquid-crystalline polymers) have been widely investigated due to their exceptional mechanical, optical and electrical properties [2,3], with their unique attribute being that LC phases can flow like liquids and can preserve the natural anisotropic structure of the glassy solid state [4,5]. This singular combination of properties is responsible for the wide range of applications that LCP presents, such as in optical data storage [6], non-linear optics [7], optical compensators [8], electronic devices [9], solid polymer electrolytes [10] and separation membranes [11]. The design and construction of membranes from LCP have aroused a great deal of interest recently owing to the importance of producing energy in a sustainable manner (artificial photosynthesis devices and fuel cells) and also for storing energy (electric batteries) [12,13,14], with the aim of trying to reproduce biological liquid-crystalline structures [15,16].

Percec and co-workers were the first to discover and examine the intramolecular self-assembly and self-organization processes of side-chain liquid-crystalline polymers (SCLCPs) into columnar structures, in which the main polymer chain is arranged into a helical inner channel surrounded by the side-chain dendrons due to an exo-recognition process of the aromatic moieties in the side-chain mesogenic groups [17,18].

In the last decade, the synthesis of side-chain liquid-crystalline polyethers and polyamines bearing different amounts of the dendron 3,4,5-tris[4-(*n*-dodecan-1-yloxy)bezyloxy]benzoate (Tap) grafted to the polymer backbone that self-assemble into a columnar structure and the subsequent preparation of membranes derived from these SCLCPs have been extensively studied by our research group [19,20,21,22]. The presence of electronegative atoms like oxygen or nitrogen in the polymeric backbone could favour interaction with cations, providing hydrophobic selective transport systems that exhibit proton permeability compared to Nafion^®^, the benchmark material in proton conductivity [23,24,25]. However, the complex self-assembly and self-organization processes of dendronized polymers can be tuned using different methods (e.g., thermal or light treatment, shearing, spinning) in order to obtain a precise channel orientation of the polymeric columns that facilitates ion transport across them [26]. As a matter of fact, several studies conducted by our research group have shown that the thermal orientation can substantially favour the self-assembly process induced by the dendronized polymeric columns, providing a more ordered structure in which an alignment of the polymeric columns perpendicular to the surface of the membrane is desired [21,22,23,27]. Therefore, an in-depth characterization of these membranes and their orientation is essential to achieve our goal, which was accomplished in recent studies by means of differential scanning calorimetry (DSC), polarized optical microscopy (POM), X-ray diffraction (XRD) and dynamic-mechanical thermal analysis (DMTA). Moreover, dielectric spectroscopy and solid-state NMR experiments have provided information on the mobility of each region of dendronized polyamines and polyethers, respectively [28,29]. These investigations focused on studying the effects that the degree of modification has on the orientation of the polymer columns and the molecular dynamics in unoriented and oriented polymer membranes, showing that the external aliphatic moieties of the side dendrons are mobile even at low temperatures, while increasing the temperature gradually increases the mobility of the central units of the polymer chains. Nonetheless, other factors, such as the length of the polymer chains, which may affect the final organization of the membrane polymer chains must also considered before carrying out the process of annealing of the membranes.

Thus, the purpose of this work was to carry out a deep investigation on how different parameters, such as grafting degree, annealing temperature, annealing time and cooling rate, influence the self-assembly and self-organization of synthesized side-chain liquid-crystalline copolyethers. These parameters were analysed in two Poly(epichlorohydrin-co-ethylene oxide) (PECH-co-EO) liquid crystalline polymers, which presented a different amount of grafted side-chain dendron methyl 3,4,5-tris[4-(*n*-dodecan-1-yloxy)benzyloxy] benzoate (Tap) in quantities of 20% (CP20) and 40% (CP40), respectively. For sake of comparison, we also analysed the features of the unmodified PECH-co-EO (CP0). The experiments performed with these liquid crystalline copolyethers show that the cooling rate from the isotropic phase to the annealing temperature and the selection of the annealing temperature play crucial roles in the orientation of the columns of both copolymers. In addition, it was found that grafting induces crystallinity in the copolymer main chain, which increases upon the orientation of the columns. In the present work, the effects of the dendron amount and of the orientation on copolymer mobility were investigated by dynamic mechanical thermal analysis (DMTA) and dielectric thermal analysis (DETA). Therefore, it is possible to understand the role of the backbone in self-assembled structures and the interaction between dendritic side groups. This information is very important to design membranes that offer a specific orientation and size of their columnar structure, and consequently to obtain good performance in operative conditions, for instance, for fuel cell applications [30].

## 2. Materials and Methods

### 2.1. Materials

Inorganic and organic compounds were purchased from Sigma Aldrich (Sigma Aldrich Química, Madrid, Spain) and Fisher Scientific (Fisher Scientific Spain, Madrid, Spain) and used as received. All the solvents were purchased from Scharlab (Scharlab, S.L., Barcelona, Spain). Potassium 3,4,5-tris[4-(*n*-dodecan-1-yloxy)bezyloxy]benzoate (Tap) was synthesized according to the previous report [22]. Poly(epichlorohydrin-co-ethylene oxide) (P(ECH-co-EO) 1:1, M_w_ = 5.01 × 10^5^, M_n_ = 1.08 × 10^5^ determined by gel permeation chromatography was purchased from Sigma-Aldrich and used as received.

**Scheme 1:**
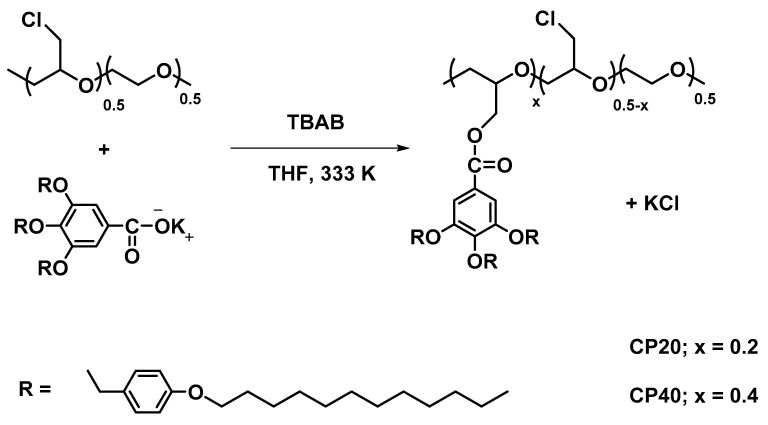
Synthesis of the copolymers.

For modification of Poly(epichlorohydrin-co-ethylene oxide) (PECH-co-EO), 0.500 g of PECH-co-EO was dissolved in 70 mL THF. Then, the stoichiometric amount of potassium methyl 3,4,5-tris[4-(*n*-dodecan-1-yloxy)benzyloxy] benzoate (Tap) was added to the copolymer. After that, stoichiometric amount of tetrabutylammonium bromide (TBAB) was added in a flask and stirred for 8 or 10 days at 333 K. The mixture was poured in ice water, and the obtained precipitate was filtered. For further purification, the precipitate was dissolved in THF and precipitated in ethanol twice to yield 2.37 g (55%) of a white product. The modification degree was calculated by the ^1^H NMR spectra of the copolymer. The mechanism of modification of the copolymer with the Tap group is shown in Scheme 1.

In this study, this chemical modification was carried out under different reaction conditions, which are given in Table 1. The copolymers were obtained with modification degrees of 20, 35, 40, respectively, which consisted of the total modification degrees of PECH-co-EO units with Tap. ^1^H NMR was used to determine the modification degree of these copolymers by comparing the normalized area value of a chemically modified region with Tap and the normalized area value of modified plus unmodified regions. In fact, 3 different modified regions (benzylic positions, aliphatic chains of the TAP and aromatic units) were used to calculate the chemical modification degrees. In all the cases the same modification degree was obtained in the 3 regions for each polymer. In this paper, we focused our attention on the copolymers modified at 20 and 40%, which were labelled CP20 and CP40, according to Scheme 1. The unmodified PECH-co-EO was labelled CP0.

### 2.2. Membrane Preparation

Membranes were prepared by the immersion precipitation method. The modified copolymer was dissolved in THF (30% *w*/*w*). After that, the homogeneous solution was cast by a casting machine (K-paint applicator, RK Paintcoat Instruments Ltd., Litlington, UK) on an FEP (fluorinated ethylene propylene) sheet support with a controlled thickness (gap size 300 μm). Then, the support, including the wet film on top, was immersed in a bath of Milli-Q water in which the polymeric membrane was formed with an asymmetric structure. After 24 h, the formed membrane was dried overnight at room temperature. The thicknesses of membranes were measured using a micrometer with a sensitivity of 2 μm.

To achieve the homeotropically oriented structure of the modified copolymer, the polymeric membrane (approx. 2 cm diameter) was placed on a hot stage (Linkam TP92, Linkam Scientific Instruments Ltd., Tadworth, UK) for the orientation process. In general, for annealing, the membrane was heated up to above its clearing temperature. Afterwards, it was allowed to cool slowly to the annealing temperature where it was kept for a variable time. After annealing, the membrane was cooled to room temperature. Finally, the uniform membrane was left at room temperature for 1h and then detached from the FEP support. In order to find the best conditions for orientation, different thermal treatment procedures were carried out, including different cooling rates (1 K/min to 0.1 K/min), annealing temperatures (363 to 380 K), and annealing times (50 h to 120 h). For scale-up, the same procedure was performed in a Hewlett Packard 5890 Series II Gas Chromatograph oven (Hewlett Packard, Palo Alto, CA, USA).

### 2.3. Characterization Techniques

X-ray diffraction measurements (XRD) were made using a Bruker-AXS D8-Advance diffractometer (Bruker Corporation, Billerica, MA, USA) with vertical theta-theta goniometer, incident- and diffracted-beam Soller slits of 2.5°, a fixed 0.5° receiving slit and an air-scattering knife on the sample surface. The angular 2θ range was between 1 and 40°. The data were collected with an angular step of 0.02° at a step/time of 0.5s. Cukα radiation was obtained from a copper X-ray tube operated at 40 kV and 40 mA. Diffracted X-rays were detected with a LynxEye-XE-T PSD detector (Bruker Corporation, Billerica, MA, USA) with an opening angle of 2.94°. Samples were placed inside a MTC-LOWTEMP chamber for in-situ temperature analysis.

The diffractograms were interpreted with the DIFFRAC.EVA 5.2 software from BRUKER.AXS, and PDF-2 database software, release 2018, from ICDD (International Center for Diffraction Data).

Calorimetric analyses were performed in aluminium standard 40 µL crucibles without pin (ME-26763) with a Mettler DSC822e thermal analyser (Mettler Toledo, Columbus, OH, USA) at a heating rate of 10 K/min with about 5 mg of sample, nitrogen as a purge gas (100 mL/min) and liquid nitrogen for the cooling system. The equipment was previously calibrated with indium (156.6 °C) and zinc (418.6 °C) pearls.

The ^1^H NMR spectra were recorded at 400 MHz on a Varian Gemini 400 spectrometer (Varian, Palo Alto, CA, USA). Deuterated chloroform (CDCl_3_) was used as a solvent and the chemical shifts were given in parts per million from TMS (Tetramethylsilane). A pulse delay time of 5 s was always used to record the ^1^H NMR spectra of the polymers.

Clearing temperatures and LC mesophases were investigated by polarised optical microscopy (POM); textures of the samples were observed with an Axiolab Zeiss (Carl Zeiss, Jena, Germany) optical microscope equipped with a Linkam TP92 hot stage (Linkam Scientific Instruments Ltd., Tadworth, UK).

DMTA measurements were performed using a TA Q800 analyzer from TA Instruments (New Castle, DE, USA) in tensile mode in rectangular films of circa 15 mm in length, 0.07 mm in thickness and 1.1 mm in width. An oscillation strain of 0.08 at 10 Hz was imposed from 123 K to 373 K at 2 K/min.

The dielectric thermal analysis (DETA) was performed using a dielectric spectrometer from Novocontrol Technologies GmbH & Co. KG, Hundsangen, Germany. The measurements were performed in the frequency range of 10^−1^ to 10^7^ Hz between 123 K to 383 K under isothermal conditions in increasing steps of 10 K. The dielectric experiments were performed in a cell constituted of two gold electrodes, where the sample electrode assembly (SEA) consisting of two stainless steel electrodes was located.

The measured dielectric relaxation spectra were fitted using the Havriliak–Negami (HN) functions, by adding as many HN functions as needed, following the proposal of Charlesworth. All the characteristic parameters of each relaxation process were determined as shown in Equation (1) [31,32,33].

(1) ε*(w)−ε∞=∑kIm[Δε{1+(iwτHNk)ak}bk]

where:

*τ_HN_* is the Havriliak-Negami relaxation time. Thus, the sub index *k* represents the number of the individual HN contributions.

*a* and *b* are parameters corresponding to the width and asymmetry broadening of the relaxation peak of the relaxation time distributions.

Δ*ε* is the value of the dielectric intensity or relaxation strength.

## 3. Results

Chemical modification of Poly(epichlorohydrin-co-ethylene oxide) (PECH-co-EO) was performed with the dendron potassium 3,4,5-tris[4-(*n*-dodecan-1-yloxy)benzyloxy]benzoate (Tap). Based on our previous results, the best results were obtained in THF at 333 K for 8 days with stoichiometric amounts of TBAB. It was shown that no improvement resulted from either changing the solvent to NMP or DMF, or increasing the Tap/chlorine molar ratio above 1:1 [19]. On the other hand, in this investigation we found that by performing the reaction for 10 days, the modification degree could be slightly increased in the case of the 1:1 Tap/chlorine molar ratio from around 70 to 80%, although the complete reaction of the nucleophile Tap could never be reached. The modification degree of the copolymers was calculated from the ^1^H NMR experiments in CDCl_3_ by comparing the areas of the aromatic peaks between 7.4 and 6.8 ppm, the benzylic proton signals at 4.8 ppm and the methylenic protons of modified units at 4.4 ppm, with the broad signal between 4 and 3.5 ppm, relative to the methylenic and methynic protons from the unmodified and modified units, as previously reported [19]. For the sake of clarity, the ^1^H NMR spectrum in CDCl_3_ of CP40 is reported in Figure S1. In this study, in order to elucidate how the Tap amount affects the final material characteristics, we focused our attention on the copolymers modified by 40 (CP20) and 80% (CP40), respectively.

### 3.1. Thermal, Microscopic and X-ray Diffraction Characterization

The copolymers under investigation, and the unmodified PECH-co-EO, were characterized by differential scanning calorimetry (DSC), polarized optical microscopy (POM) and X-ray diffraction (XRD), in order to establish the presence or absence of crystallinity and/or liquid crystallinity. In the case of CP20 and CP40, DSC and XRD characterizations were also performed on the membranes oriented as described below.

The results of calorimetric analysis are shown in Table 2.

In the case of the unmodified CP0, only the glass transition could be evidenced in the DSC thermogram, at around 241 K. Nevertheless, the presence of some crystallinity cannot be totally ruled out. For instance, Jin et al. reported on amorphous blends of poly (methyl methacrylate)/poly (ethylene oxide). They found some difference between the calculated composition from NMR and the known bulk composition that suggested the presence of ~5 wt % phase-separated PEO in addition to a mixed amorphous phase and postulated that the phase-separated PEO arose from the constrained and crystalline units, but no PEO crystallinity was detected by DSC [34].

**Figure 1:**
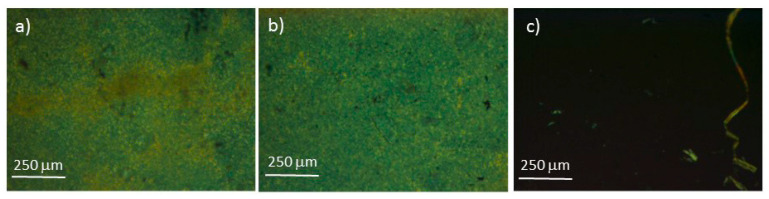
Optical micrographs under crossed polarizers of CP40 at 300 K (**a**), 335 K (**b**) and 433 K (**c**).

The glass transition temperature was shown to increase as a consequence of the copolymer modification with Tap. This could be reasonably expected, based on the restrictions on main chain mobility determined by the steric hindrance of this moiety. On the other hand, CP20 and CP40 showed two endotherms, which, according to POM observation and XRD experiments, could be attributed to the melting of the main chain and the clearing of the LC phase, respectively. Figure 1 shows optical micrographs under crossed polarizers of CP40 at various temperatures.

At 300 K and 335 K, a birefringent texture characteristic of a liquid crystalline phase, could be observed by POM (Figure 1a,b). The differences between the patterns observed at these temperatures are difficult to describe. Nevertheless, birefringence completely disappeared above 383 K, thus suggesting that the endotherm observed at this temperature in DSC corresponds to the clearing of the LC phase. The clearing temperatures found for CP20 and CP40 are in agreement with those determined for the copolymers from the same family that have been previously reported [19]. In our previous investigation, we could not evidence any crystallinity from XRD and the presence of a crystalline phase was suggested by the presence of two relaxation processes in the ^13^C NMR spectrum of the methylenic carbon of the modified units.

**Figure 2:**
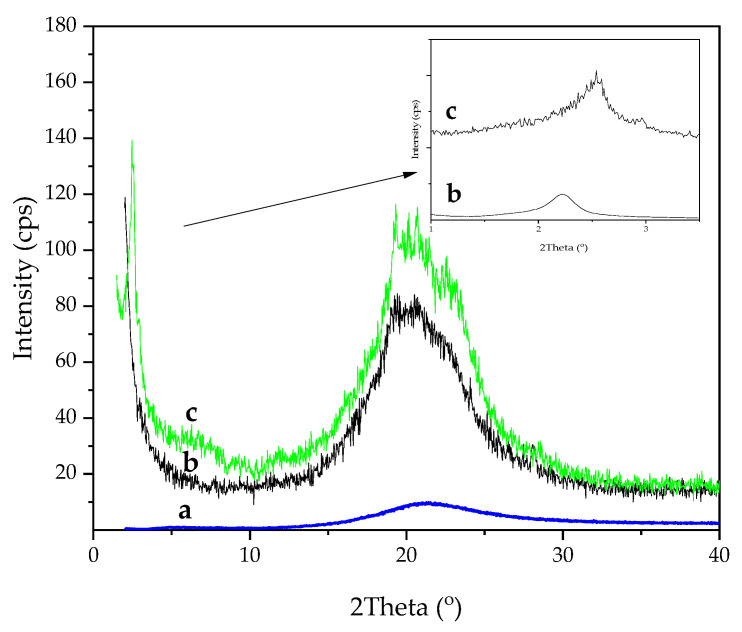
WAXD at room temperature of: CP0 (**a**), CP40 (**b**) and CP20 (**c**). Inset: SAXD of CP40 (**b**) and CP20 (**c**).

Figure 2 demonstrates the wide angle (WA) XRD pattern of CP0, CP20 and CP40 at room temperature.

**Figure 3:**
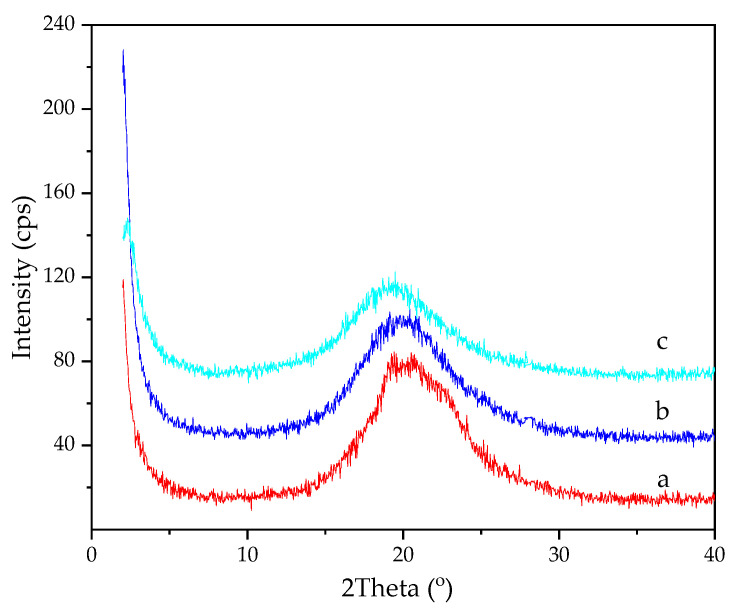
WAXD of CP40 at: 293 K (**a**), 323 K (**b**) and 393 K (**c**).

As far as CP0 is concerned, the XRD pattern only showed a halo around 2θ = 20°, indicative of an amorphous phase. This sample was annealed at 328 K for variable time, but no change could be detected in its XRD pattern. CP20 and CP40 exhibited a more intense, clearly asymmetric halo around 2θ = 20°, which suggests the presence of some crystalline reflections approximately between 2θ = 18° and 24°. This asymmetry vanished when the sample was heated to 323 K, as shown in Figure 3 for CP40.

This diffraction angle region also coincides with the WAXD pattern of crystalline poly (ethylene oxide) [35]. Therefore, it can be reasonably concluded that the melting observed in CP20 and CP40 cameomes from a main chain partial crystallization of the polyether chain.

From the calorimetric data, as an approximation, we could roughly estimate the degree of crystallinity Xc of the copolymers using the following equation:

(2) $$X_{c}=\frac{\Delta H_{m}}{\Delta H_{m100}}\times 100$$

where ΔH_m_ is the experimental melting enthalpy value and ΔH_m 100_ is the reported melting enthalpy for 100% crystalline Poly(ethylene oxide), used as a reference [36]. The resulting values are shown in Table 2. Although the calculated absolute values of crystallinity are strongly affected by the approximations performed, the comparison among the different systems showed interesting evidence. While the unoriented CP40 and CP20 exhibited similar Xc and melting temperature values, both samples obtained after the orientation of the columns, as described below, exhibited increased melting temperatures and crystallinity degrees over the unoriented copolymer. This effect was much more evident for CP40, which had the highest modification degree. Therefore, it can be reasonably concluded that the presence of the dendrons is able to induce order in the copolymer main chain, and this effect is more evident when they are oriented. A certain order in the main chain induced by side modification was also evidenced by Reina et al. in PECH-co-EO modified with 10-undecenoate and 4-pentenoate [37]. The effect found in CP20 and CP40 was more deeply investigated by DMTA and DETA analyses, as reported below.

Concerning the XRD pattern in the small angle region (SAXD), a sharp reflection at 2θ = 2.2° (CP40) and 2θ = 2.3° (CP20), respectively, could be seen (Figure 2, inset), which was compatible with the presence of a columnar mesophase. The corresponding spacing of 40 Å and 38 Å, respectively, corresponded to the (100) plane and can be attributed to the distance between columns, similarly to our previously investigated systems [19,22,23,38,39]. The diffuse halo around 2θ = 20° was related to the distance between disks. From these values, we could calculate the dimension of the unit cell (parameter a), which was about 46 Å for CP40 and 44 Å for CP20, respectively.

As far as the mesophase stability of the oriented membranes is concerned, when determined by DSC (Table 2), we found no difference in the clearing temperature and a slight decrease of the clearing enthalpy for CP40, while in the thermogram of the oriented CP20, neither Tg nor the peak corresponding to the clearing could be detected, and the clearing temperature was detected by POM. Therefore, the clearing enthalpy could not be calculated.

### 3.2. Optimization of the Orientation Procedure

As previously stated, the use of membranes based on columnar polymers for cation transport requires the proper orientation of the columns for them to work as ion channels. For this purpose, we based our analysis on the findings of Percec et al., who stated that the columnar self-assembly of dendronized polymers is driven by the aromatic moieties, which induce a helical arrangement. Therefore, these materials can be homeotropically oriented by slowly cooling them from the molten state to room temperature, due to the π-π stacking of aromatic moieties [30]. Indeed, we could successfully apply this approach to several systems of our synthesis, based on dendronized polyethers or polyamines [20,21,22,23,24,25,27]. In a previous paper, we investigated the influence of the used support on the final polymer orientation [23], and found that it does not play a crucial role, which seems to indicate that the hierarchical structures of these dendronized copolymers tend to form down from the top surface. It must be remembered that the aliphatic tails present in the dendrons possess high mobility and low surface energy. Therefore, their higher air compatibility and entropy may favour their movement toward the air interface over the main chain components. Consequently, one can suppose the anchoring of the dendrons to the air interface. An analogous behaviour was recently found in the case of the nanocylinder orientation of a liquid crystal block of copolymer film based on poly(ethylene oxide) and a polymethacrylate bearing azobenzene mesogen side chains [40]. In the end, we chose FEP as a convenient support for membrane preparation and thermal orientation, since they could be easily peeled off.

In our previous studies [21,22,23,27], we could get satisfactory orientation of the mesogenic columns, which led to promising transport properties; nevertheless, we did not examine the influence of different parameters, i.e., annealing temperature, cooling rate and annealing time, on the final degree of orientation. In this paper, we tried to optimize the procedure for the thermal orientation of CP20 and CP40. Given the similarities of the two systems in the initial clearing temperatures and crystallinity degrees, we fine-tuned the thermal treatment on CP40 membranes and successfully applied the same procedure to CP20. In this regard, four procedures were carried out:

- For sample CP40 (a), the membrane was heated up (10 K/min) to 398 K, then it was cooled slowly (1 K/min) to 363 K where it was kept for 50 h and subsequently cooled to room temperature at a rate of 10 K/min.
- For sample CP40 (b), the membrane was heated up (10 K/min) to 413 K, then it was cooled slowly (1 K/min) to 378 K where it was kept for 50 h. In the end, the membrane was allowed to cool (10 K/min) to room temperature.
- In the case of sample CP40 (c), the membrane was heated up (10 K/min) to 413 K, then it was cooled slowly (0.1 K/min) to 378 K where it was kept for 69 h. Finally, the membrane was allowed to cool (10 K/min) to room temperature.
- For sample CP40 (d), the membrane was heated up (10 K/min) to 413 K. It was kept at the same temperature for 30 min. Then it was cooled slowly (0.1 K/min) to 380 K where it was kept for 120 h. Finally, the membrane was allowed to cool to room temperature at 10 K/min.

**Figure 4:**
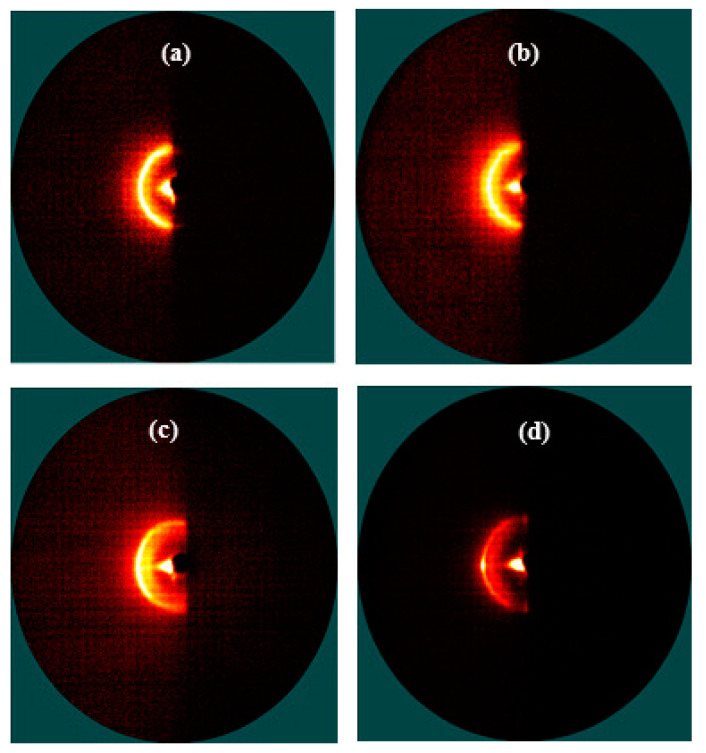
Debye ring pattern of CP40 (samples (**a**–**d**)) after the thermal treatment with different conditions.

As a parameter indicating the achieved columnar orientation, we chose the width at half height (WHH) of the peak obtained from the azimuthal scan of the reflection at 2θ = 2.2° (corresponding to the intercolumnar distance). The different procedures applied are summarized in Table 3. The resulting WHH values and Debye rings of the XRD patterns indicative of the achieved orientation are shown in Table 3 and Figure 4, respectively. The intensity versus azimuthal angle graphs are shown in Figure S2.

As it is evident from Figure 4a,b, samples CP40 (a) and CP40 (b) show no column orientation at all, as demonstrated by the absence of any polarization of the 2θ reflection at 2.2°. On the other hand, for sample CP40 (c) this reflection exhibits some polarization (Figure 4c), giving a WHH = 92°. In CP40 (d) it lies in the equator (Figure 4d), which corresponds to a diffraction in the membrane plane. In this last case, the WHH was 8.3°, indicating that the intercolumnar distance was mainly lying in this plane. The peak maximum in the azimuthal scan as located at 90° in both CP40 (c) and (d), confirming that a homeotropic orientation of the columns can be achieved, although it was just partially in the case of CP40 (c). Given this evidence, one can draw the following conclusions with regards to the orientation procedure:

- The cooling rate from the isotropic phase to the annealing temperature seems to play a crucial role: the lower the rate, the better the orientation.
- The annealing temperature should be as close as possible to the clearing point determined by DSC. In this regard, given the higher mobility of the copolymer at higher temperatures, one has to increase the annealing time accordingly, i.e., we passed from 69 h in the case of T_clearing_ − T_annealing_ = 5 K (CP40 (c)), to 120 h for T_clearing_ − T_annealing_ = 3 K (CP40 (d)). It must be also pointed out that when CP40 was annealed at 378 K (T_clearing_ − T_annealing_ = 5 K) for longer times, no improvement in column orientation was achieved.

### 3.3. Dynamic-Mechanical Thermal Analysis (DMTA)

The modified copolymers CP20 and CP40, and the unmodified CP0, were further investigated by dynamic mechanical thermal analysis (DMTA). Changes in the complex moduli and peaks in the tanδ curve can be used to identify the amount of crystallinity and/or liquid crystallinity as it affects the transition temperatures (Tg and Tm), and the viscoelastic properties. In the case of CP20 and CP40, a DMTA characterization was also performed on the oriented membranes.

**Figure 5:**
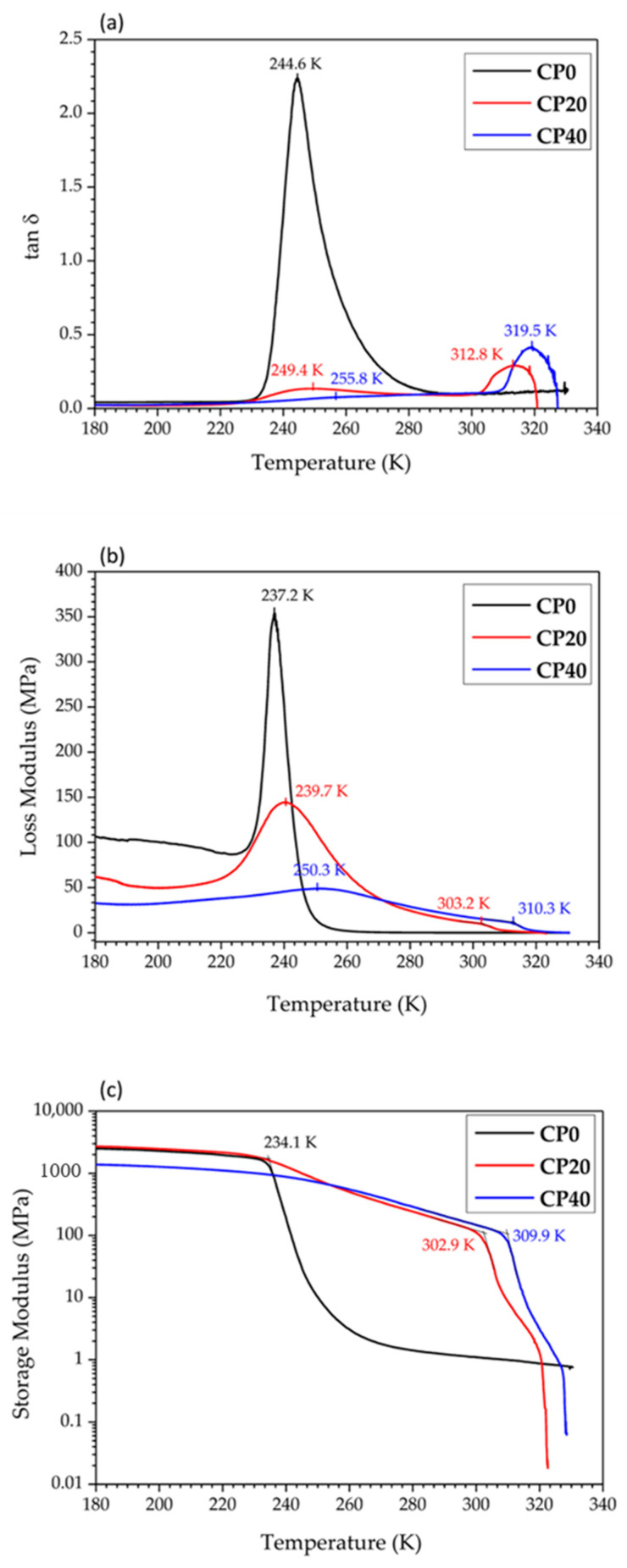
DMTA analysis for CP0, CP20 and CP40 samples. (**a**) Tanδ, (**b**) loss modulus and (**c**) storage modulus evolution with temperature.

In Figure 5, significant differences can be observed in the DMTA behavior between unmodified CP0 and the modified CP20 and CP40 samples. The tanδ peak (Figure 5a) in sample CP0 is sharp and high, but shifts to higher temperature, decreases in amplitude and significantly widens as the degree of modification increases. This effect is similar but more evident in the loss modulus peaks (Figure 5b) located at around 237 K for CP0, 238 K for CP20 and 250 K for CP40. The degree of crystallinity has the effect of limiting the motion of the main chain, which becomes more mobile as it is heated up through its glass transition temperature. Since the glass transition is due to the increased movement of the amorphous phase, an increase in the degree of crystallinity produces an increase in the Tg, together with a widening of the transition, because more thermal energy is needed for the main chain to become mobile and able to dissipate energy. The increase in the degree of modification was also evidenced by a slight increase in the melting temperature of the main chain detected with a small peak in the tanδ curve or a sharp decrease in loss modulus from 314 K for CP20 to 319 K for CP40. Concerning the storage modulus evolution (Figure 5c) the behavior in the glass transition region was similar to the loss modulus or tanδ, although less evident. For the CP0 sample, the drop of the storage modulus in the transition region was sudden and sharp, but this decrease changed to a more progressive and smooth one as the degree of modification increased, being almost inappreciable in CP40. Contrarily, the melting point in samples CP20 and CP40 was evidenced by a sharp drop as the crystalline structure approached the melting point. Both transition temperatures (Tg and Tm) obtained from DMTA were in accordance with those obtained from DSC and shown in Table 2.

**Figure 6:**
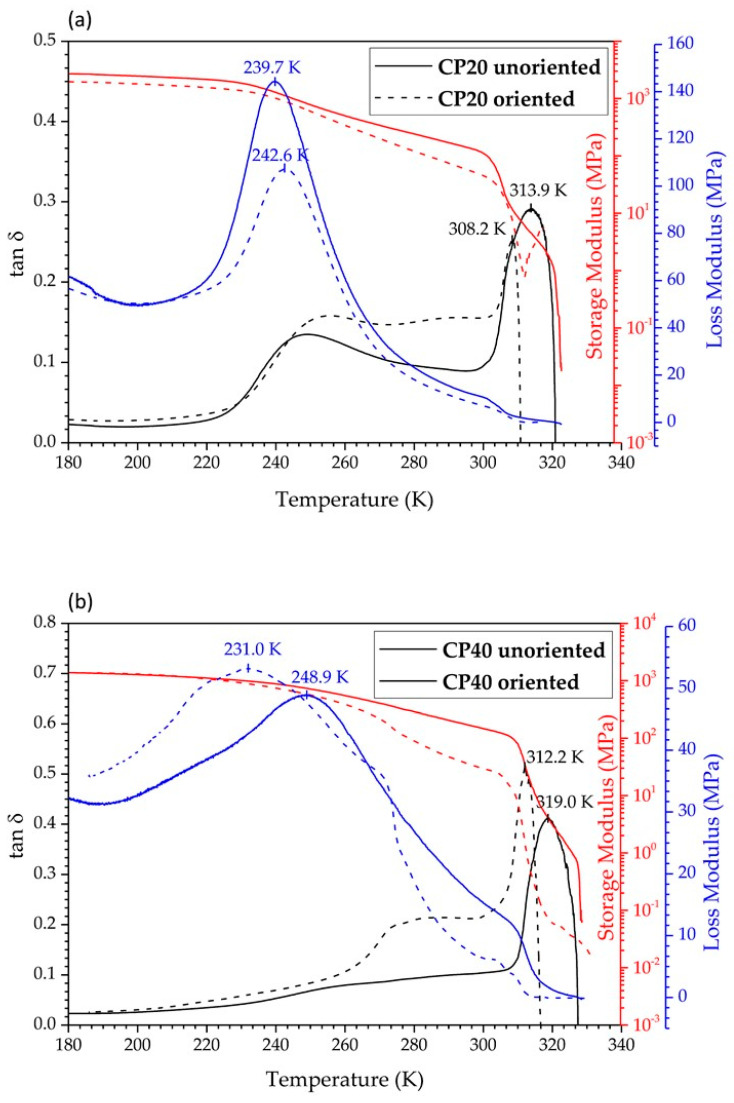
Comparison of DMTA analyses for the CP20 (**a**) and CP40 (**b**) oriented and unoriented samples.

DMTA experiments were also performed in the oriented samples, and the results can be seen in Figure 6. As a general trend, in the oriented samples, the thermomechanical behaviour became less evident and defined, and it was difficult to detect the transition zones at high temperatures, especially for CP40. When comparing the peak of the loss modulus (and tanδ peak) of the oriented and unoriented samples, no appreciable changes ere evidenced in Tg for sample CP20 (Figure 6a) with only a slight decrease in the height of the tan δ peak for the oriented peak. On the contrary, for sample CP40 (Figure 6b) a change seemed to appear in the temperatures of the loss modulus and the tanδ peaks, and in the shape of the curve one could observe a decrease in Tg, but the curves looked wider and less defined. The melting temperature also seemed to decrease in both samples but the signal was difficult to detect and no proven conclusions could be obtained.

### 3.4. Dielectric Thermal Analysis (DETA)

**Figure 7:**
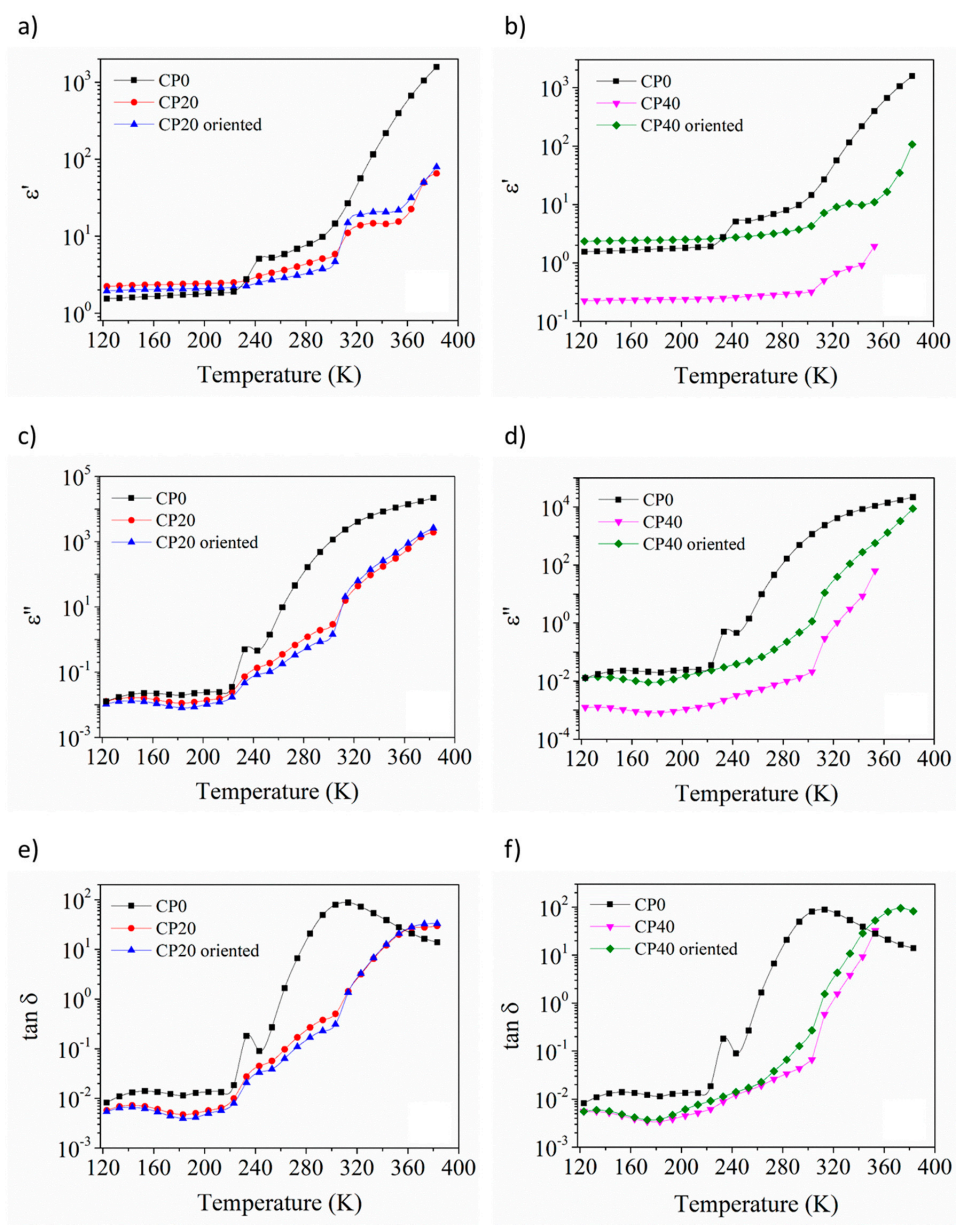
(**a**,**b**) Real part of the complex permittivity; (**c**,**d**) imaginary part of the complex permittivity; (**e**,**f**) loss tangent of the unmodified copolymer (CP0) of the modified oriented and unoriented copolymers CP20 and CP40, respectively, for a frequency of 10 Hz.

The dielectric relaxation spectra of unoriented and oriented modified copolymers CP20 and CP40, and the unmodified CP0, were obtained in terms of the real and imaginary parts of the complex dielectric permittivity and the loss tangent angle. Figure 7 plots the isochronal curves of all these properties as a function of the temperature range (123 to 383 K), respectively, for a frequency of 10 Hz. The dielectric relaxation spectrum of these copolymers is much more complex than the mechanical spectrum. At least three Havriliak–Negami functions related to intra or inter-molecular motions were observed.

The dielectric spectra of the unmodified copolymer (CP0) are composed of four dielectric relaxations, as shown in Figure 7. More precisely, two dielectric relaxations were located at low temperatures and two relaxations were located at mid to high temperatures, in an increasing temperature order. The first dielectric relaxation appeared in the range of 123 K to 173 K at a frequency of 10 Hz. At these low temperatures, the molecular arrangements are of intramolecular origin, and only local modes of mobility can be attributed as the origin of this relaxation. In this case, this relaxation can be assigned to local motions in the amorphous part of the PEO segments [41].

A second dielectric relaxation occurred between 163 K and 223 K at a frequency of 10 Hz. This is a very complex zone that other authors have attributed to the overlapping of two types of motions. One is the γ’ relaxation, which is similar to the γ, but it occurs in the amorphous zone, hindered by the lamellae that are part of the crystalline domains [41]. In our case, as the work previously referenced, the γ’ relaxation appears to be overlapped with the β relaxation. Though no clear evidence of crystallinity was found for CP0, a certain local order cannot be ruled out. Therefore, one can postulate the existence of amorphous zones but with restricted mobility, the relaxation of which can be evidenced by the higher sensitivity of DETA. The other relaxation is attributed to the cooperative motion of the amorphous region of the PEO segments. For this reason, this relaxation could be labelled as a β relaxation because it is a cooperative movement but of only one part of the copolymer, or an α _PEO_ relaxation to define exactly the nature of the molecular movement.

The next molecular relaxation was found in the temperature range between 223 K and 248 K at a frequency of 10 Hz. As other researchers have discussed, this dielectric relaxation is attributed to the cooperative motion of the PECH segments, which would already affect the whole main chain. Thus, this relaxation was considered as the glass transition Tg, and therefore it is called the α relaxation [28,29,42,43]. This peak would be related to the maximum found in the spectrum of mechanical relaxations in the same temperature range.

Additionally, between 253 K to 383 K another dielectric relaxation was found, whose maximum peak value was found at 323 K at a frequency of 10 Hz. The temperature at which this relaxation appeared fully coincides with the values observed by Silva et al. for the crystallization peak followed by the melting of PECH-co-EO [43]. These authors suggest that the crystalline phase is probably formed by the organization of the ethylene oxide blocks present in the copolymer structure. Therefore, this relaxation can be attributed to a molecular motion that promoted the melting transition.

Figure 7 also shows the dielectric spectra of the copolymers CP20 and CP40, which are composed of four molecular relaxations. However, not all of them have the same molecular origin as those of CP0.

At the lowest temperature, from 123 K to 173 K at a frequency of 10 Hz, a relaxation appears. Note that this relaxation appears at lower temperatures than the γ relaxation found in CP0. These results are in accordance with the molecular mobility found, for instance, in the dielectric spectra of PECH40 and PECH80 [29], as well as in PAZE100 and PAZE40 [28]. Thus, this relaxation can be ascribed to an intramolecular motion related to the benzyloxy terminal group of the dendrimer side groups.

**Figure 8:**
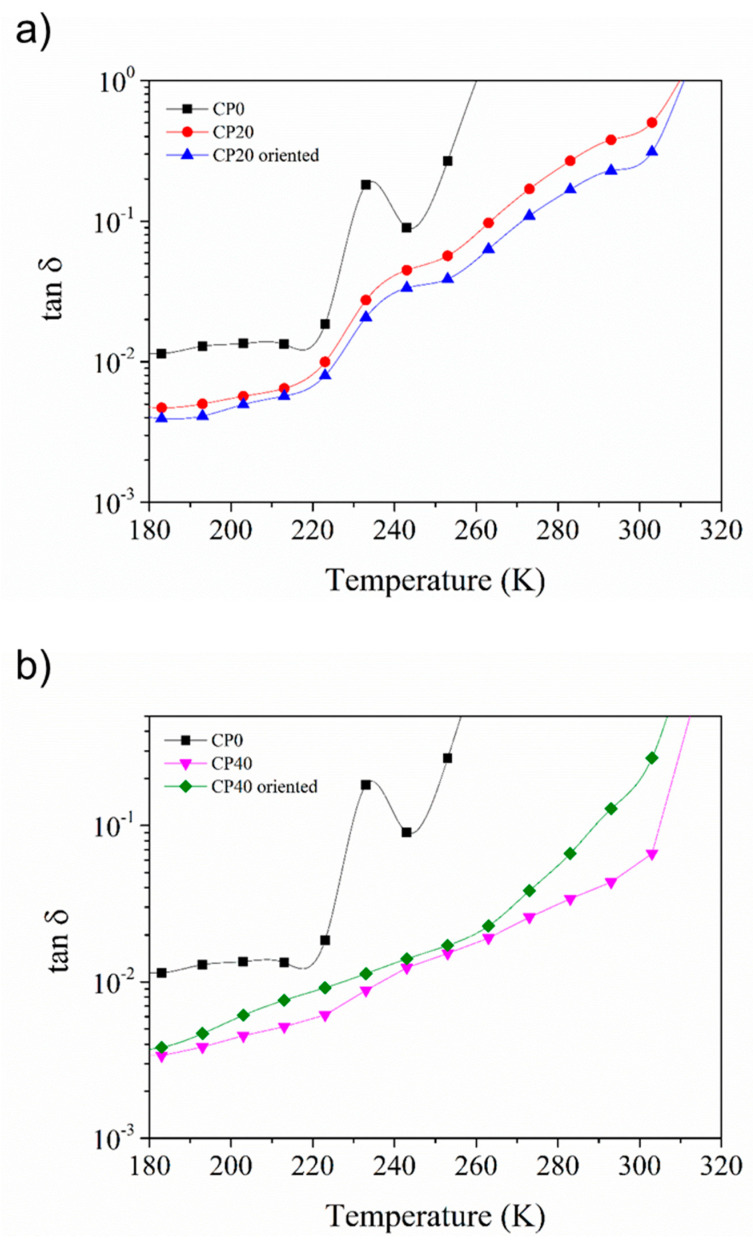
Tan δ of the: (**a**) unmodified (COP0) and modified oriented and unoriented copolymer CP20; and (**b**) modified oriented and unoriented copolymer CP40, respectively, for a frequency of 10 Hz.

The second relaxation, from 163 K to 223 K at a frequency of 10 Hz, coincided with the molecular arrangements observed in the copolymer (COP0) in the same temperature interval. Therefore, it was related to the motion of the PEO segments. The intensity of this relaxation was lower since the dendritic chains hindered this motion. Figure 8 displays a comparison between the dielectric spectra of the oriented and unoriented CP20 and CP40, respectively. The orientation process did not produce any significant variation in the dielectric spectra of either copolymer, regardless of the number of dendronized units. Indeed, the same molecular mobility was observed, and only slight shifts towards higher temperatures were found. This is to be expected due to the high level of restrictions imposed by the orientation process. The trend observed in the dielectrical plots (Figure 8a,b) is similar to that observed in the DMTA results (Figure 6a,b). There are no appreciable differences between the unoriented and oriented CP20 samples which both present similar shapes in the evolution of the tanδ. Contrarily, for the oriented sample CP40, the tanδ curve slightly differed from its unoriented counterpart and presented a shape more undefined, with no significant changes in the slope, and without a clear peak in the transition zone, as was also observed in the DMTA curves.

The third relaxation, as Figure 7 indicates, from 223 K to 273 K at a frequency of 10 Hz, is attributed to the glass transition of the copolymers. The intensity of this relaxation decreased progressively going from CP20 to CP40, which is in agreement with the increase of cristallinity consequent to the side modification of the copolymer.

Finally, in the same Figure 7, the last broad relaxation at temperatures higher than 348 K at a frequency of 10 Hz corresponded to two overlapping processes, i.e., the melting of the copolymer main chain (initial segment of the transition) and the clearing (end segment of the transition) of the copolymer liquid crystalline phase.

It is possible to assume that the differences observed between the different copolymers in this higher temperature range were mainly due to the melting process. Figure 8 shows that, at lower temperatures there are differences between the copolymers due to their different degree of crystallinity. Therefore, these differences also appear during the melting process and are more evident in the case of CP40.

## 4. Conclusions

PECH-co-EO was modified with the Dendron 3,4,5-tris[4-(*n*-dodecan-1-yloxy)benzyloxy]benzoate (Tap), at two different extents, producing two copolymers with 20% (CP20) and 40% (CP40) dendronized repetitive units, respectively. Both copolymers exhibited a liquid crystalline columnar mesophase. Moreover, the presence of the Dendron induced a partial crystallization of the main chain with respect to the unmodified PECH-co-EO (CP0), probably involving the ethylene oxide units. Membranes were prepared out of these copolymers, and a proper orientation of the columnar channels was achieved by means of a thermal treatment. A fine-tuning of this procedure revealed that the cooling rate and the annealing temperature were crucial parameters to obtain the highest homeotropic orientation.

Copolymer mobility was investigated by DMTA and DETA in both the unoriented and oriented membranes. Mechanical tests evidenced that grafting with the dendron strongly hindered copolymer motions, as expected, such that the higher the modification degree, the higher the degree of crystallinity, Tg and melting temperature; that is, the copolymer needed more thermal energy for the main chain to become mobile and dissipate energy. A DETA analysis showed four molecular relaxations in both the unmodified and modified copolymers, although some of them had different origins. In the case of CP0, the unmodified copolymer, we could distinguish local motions of the amorphous PEO portions and relaxations of chain portions with somewhat restricted mobility, the glass transition, and finally a molecular motion that promoteed the melting transition. On the other hand, CP20 and CP40 showed an intramolecular motion related to the benzyloxy terminal group of the dendrimer side groups, and another relaxation related to the motion of the PEO segments, which had lower intensity than in CP0, which we attributed to the presence of the dendritic moieties. At higher temperature, we could distinguish the glass transition and the partially overlapped main chain melting and liquid crystalline phase clearing. The analysis of the dielectric spectra of these copolymers was quite complex and intriguing. Therefore, it will be deepened in a forthcoming paper.

Neither DMTA nor DETA evidenced great differences between the unoriented and oriented membranes, regardless of the quantity of dendrons. Indeed, we observed the same molecular mobility and slight shifts towards higher temperatures as expected, on the basis of the restrictions imposed by the orientation process.

The differences in the modification degree of the copolymers through the introduced crystallinity and modified molecular mobility will surely affect their performances as membranes. Therefore, in part 2 of this paper we report on the membrane characterization and their transport properties.

## Figures and Tables

**Table 1:** Reaction conditions and resulting modification degrees and yields for the P(ECH-co-EO) modification with Tap.

Sample	Chlorine/Tap (mol/mol)	PECH-co-EO (mol·10^−3^)	Tap (mol·10^−3^)	Time (days)	T(K)	Modification Degree of PECH-co-EO (%)	Yield (%)
CP20	1:0.5	7.4	3.7	8	333	20	75
CP35	1:1	3.7	3.7	8	333	35	55
CP40	1:1	3.7	3.7	10	333	40	65

**Table 2:** Calorimetric characterization of CP0, CP20 and C40. Glass transition (Tg), melting (T_m_) and clearing (T_cl_) temperatures; melting (ΔH_m_) and clearing (ΔH_cl_) enthalpies per mol repetitive unit; crystallization degree (Xc). Data relative to unoriented samples determined from DSC second heating scan.

Sample	Tg (K)	T_m_ (K)	ΔH_m_ (kJ/mol)	Xc (%)	T_cl_ (K)	ΔH_cl_ (kJ/mol)
CP0	241	-	-	-	-	-
CP20	262	308	2.79	32	378	0.061
CP20 oriented	n.d.	312	3.27	38	377 *	n.d.
CP40	262	308	2.56	30	383	0.17
CP40 oriented	258	314	4.78	52	383	0.14

* From POM observation, n.d.: not determined.

**Table 3:** Different thermal treatments and resulting peak width at half height (WHH) and angle of orientation of annealed membranes of the CP40 sample.

Sample	Annealing Temperature (K)	Cooling Rate (K/min)	Annealing Time (hours)	WHH (°)	Angle of Orientation (°)
CP40 (a)	363	1	50	No orientation	-
CP40 (b)	378	1	50	No orientation	-
CP40 (c)	378	0.1	69	92	90
CP40 (d)	380	0.1	120	8.3	90

## Data Availability

The data presented in this study are available on request from the corresponding author. The data are not publicly available due to their deposition at an offline disk, MEMTEC group, URV, Tarragona, Spain.

## Supplementary Materials

The following are available online at https://www.mdpi.com/article/10.3390/polym13203532/s1, Figure S1: 1H NMR spectrum in CDCl3 of CP40 at room temperature, Figure S2: Phi diffractograms from azimuthal scan on the reflection at 2θ = 2.2° of CP40 (samples (a)–(d)) after the thermal treatment with different conditions.
