# Supplementary material for: Membranes for Cation Transport Based on Dendronized Poly(epichlorohydrin-co-ethylene oxide). Part 1: The Effect of Dendron Amount and Column Orientation on Copolymer Mobility
Source: Polymers (Basel). 2021 Oct 14;13(20):3532. doi: 10.3390/polym13203532 (PMC8540024; doi:10.3390/polym13203532)
Supplement: Supplementary file 1 [file polymers-13-03532-s001.zip › polymers-1417783-supplementary.pdf]

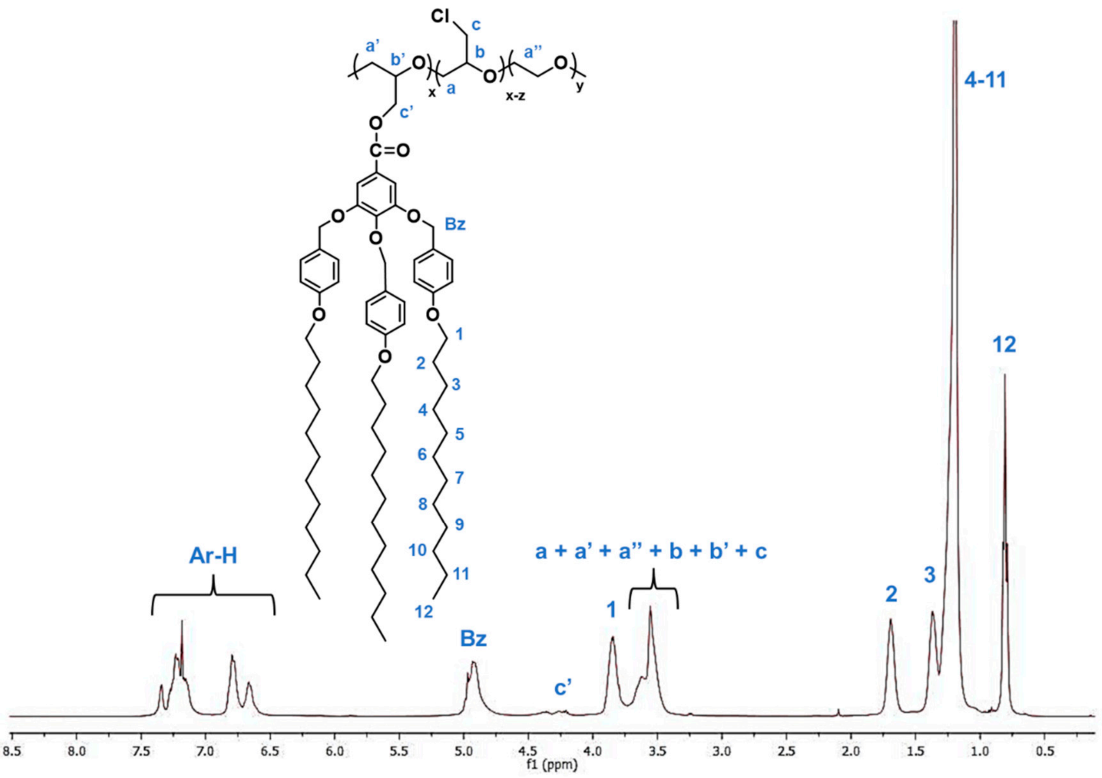

Figure S1. ^{1}H NMR spectrum in $CDCl_3$ of CP40 at room temperature.

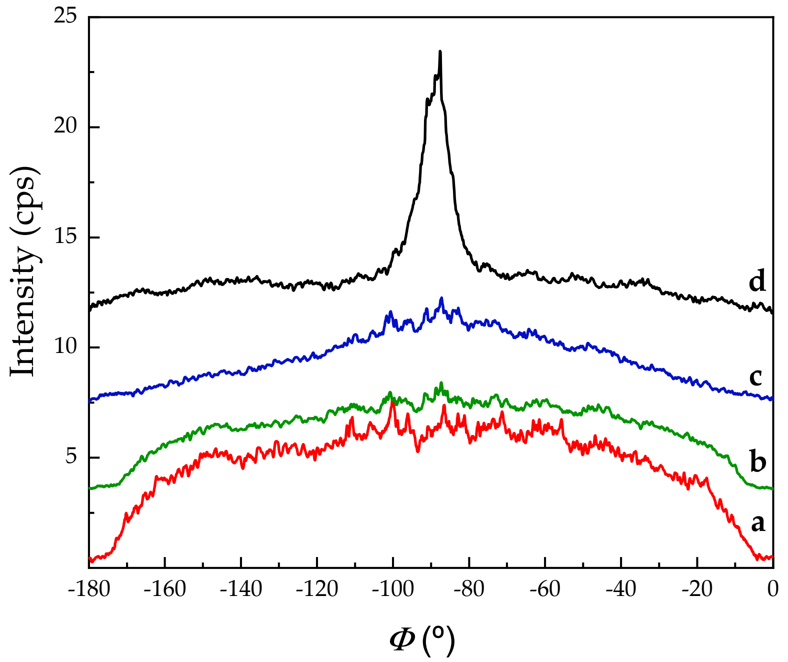

Figure S2. Phi diffractograms from azimuthal scan on the reflection at 2θ = 2.2° of CP40 (samples (a) – (d)) after the thermal treatment with different conditions.
